# Pulmonic Valve Repair in a Patient with Isolated Pulmonic Valve Endocarditis and Sickle Cell Disease

**DOI:** 10.1155/2015/732073

**Published:** 2015-06-23

**Authors:** Timothy Glew, Migdalia Feliciano, Dennis Finkielstein, Susan Hecht, Daryl Hoffman

**Affiliations:** ^1^Department of Cardiology, Mount Sinai Beth Israel, New York, NY 10003, USA; ^2^Department of Medicine, Mount Sinai Beth Israel, New York, NY 10003, USA; ^3^Department of Cardiothoracic Surgery, Mount Sinai Beth Israel, New York, NY 10003, USA

## Abstract

A 49-year-old woman with sickle cell disease presented with one month of exertional dyspnea, weakness, and fever and was diagnosed with isolated pulmonic valve endocarditis secondary to methicillin-resistant *Staphylococcus* bacteremia in the setting of a peripherally inserted central venous catheter. Chest computerized tomography showed multiple bilateral pulmonary nodular opacities consistent with septic emboli. Transthoracic and transesophageal echocardiograms revealed a large echodensity on the pulmonic valve requiring vegetation excision and pulmonic valve repair. In conclusion, isolated pulmonic valve endocarditis is a rare cause of infective endocarditis that warrants a high index of clinical suspicion. Furthermore the management of patients with sickle cell disease and endocarditis requires special consideration.

## 1. Introduction

Pulmonic valve infective endocarditis is uncommon, accounting for less than 1.5–2% of patients diagnosed with infective endocarditis [1]. Isolated pulmonic valve endocarditis even in the presence of structural heart disease is rare, with fewer than 90 cases previously reported. Risk factors for right sided endocarditis include intravenous drug abuse and central venous catheter or pacemaker implantation. We describe the first reported case of isolated pulmonic valve endocarditis requiring pulmonic valve repair in a patient with sickle cell disease.

## 2. Case Description

A 49-year-old African American woman with past medical history of sickle cell disease (Hgb SS) hypertension and transient ischemic attack was admitted to a community hospital for evaluation of shortness of breath, dyspnea on exertion, weakness, and fever of one-month duration.

Four months prior to admission the patient was found to have methicillin-resistant* Staphylococcus aureus* (MRSA) bacteremia for which a peripherally inserted central catheter (PICC) line was placed for long term intravenous (IV) vancomycin. A transthoracic echocardiogram (TTE) done at that time showed no evidence of endocarditis or significant valvular disease. Surveillance blood cultures were negative, she was transitioned to oral rifampin, and her PICC line was removed.

On the index admission to a community hospital, she presented with shortness of breath and fever to 102.4°F. A complete blood count showed leukocytosis (38,000 cells/mm^3^), 92% neutrophils, and a microcytic anemia (Hgb 8.2 g/dL). She was found to be in sickle cell crisis. Two sets of blood cultures drawn on the day of admission grew MRSA and she was started on a sepsis protocol with intravenous vancomycin and aztreonam. An electrocardiogram (ECG) obtained revealed sinus tachycardia with nonspecific T wave abnormalities in the inferior leads. Her chest X-ray showed bilateral reticulonodular opacities in the mid to lower lung fields. A TTE report from day four at the community hospital revealed a normal left ventricular ejection fraction, no significant valvular disease, and no obvious vegetation.

Ten days after admission the patient developed respiratory distress and required endotracheal intubation and mechanical ventilation. Chest computerized tomography (CT) showed multiple bilateral pulmonary nodular opacities, with cavitations consistent with septic emboli. The patient was stabilized and transferred to our hospital for further management.

**Figure 1:**
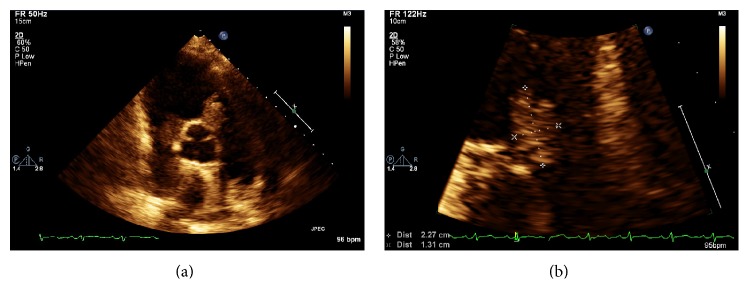
Transthoracic echocardiogram parasternal short axis showing a 2.2 × 1.31 cm echodensity on the pulmonic valve.

**Figure 2:**
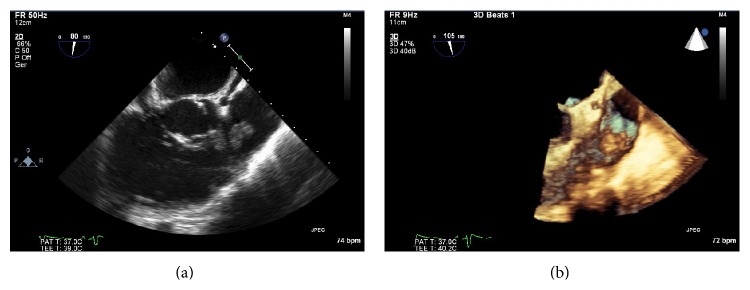
Transesophageal echocardiogram with a 2.4 × 1.8 cm echodense mobile mass seen on the pulmonic valve consistent with large vegetation in 2D and 3D views.

On admission to our hospital she was afebrile and had a previously undocumented 2/6 diastolic murmur over the pulmonary area, a leukocyte count of 15,900 cells/mm^3^, and 75% neutrophils. A TTE was obtained which showed large vegetation on the pulmonic valve (2.3 × 1.3 cm) and trace pulmonic regurgitation (Figures 1(a) and 1(b)). She was continued on vancomycin and rifampin and repeat blood cultures were negative. Given the large size of the vegetation a transesophageal echocardiogram (TEE) was obtained for potential surgical planning. The TEE revealed a 2.4 × 1.8 cm echogenic mobile mass on the pulmonic valve, trace pulmonic regurgitation, and no other significant valvular pathology (Figures 2(a) and 2(b)).

At this time a multispecialty heart valve team was assembled that included cardiology, cardiothoracic surgery, cardiac anesthesiology, infectious disease, and hematology. The consensus was for a trial of conservative management; however the patient had persistent leukocytosis and ongoing hemodynamic instability suggesting that antibiotic penetration might be limited. Ultimately the heart team recommended surgery. Her course was further complicated by respiratory distress requiring reintubation and surgery was delayed. Given her history of sickle cell disease an exchange transfusion was planned prior to surgery to reduce the percentage of Hemoglobin S but ultimately was not needed as preoperative transfusion corrected her anemia and reduced the HbS percentage to 28.7%.

**Figure 3:**
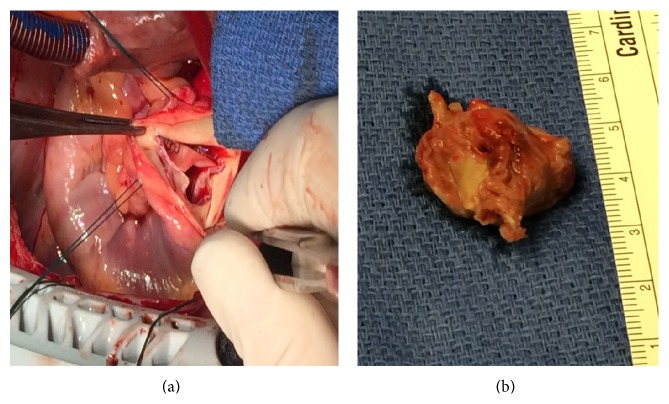
The pulmonic valve in vivo (a) and the excised valve leaflet (b).

Twenty-five days after her initial presentation she underwent pulmonic valve repair. While on cardiopulmonary bypass normothermia was maintained and 2.5 × 2 cm pulmonic valve vegetation was excised en bloc with the left cusp of the pulmonic valve (Figures 3(a) and 3(b)). The resected leaflet was replaced with glutaraldehyde treated bovine pericardial patch shaped to match one of the other pulmonic cusps and sewn into place along the annulus with a continuous polypropylene suture. The excised pulmonic valve vegetation was sent to pathology and revealed bacterial colonies in fibrinous exudates, consistent with bacterial endocarditis.

The patient was weaned off bypass with no evidence of pulmonic regurgitation on intraoperative TEE. The patient was extubated soon after surgery and had an uneventful postoperative course. Repeat postoperative blood cultures were negative. A postoperative TTE revealed a normal left ventricular size and systolic function and normal appearance of the pulmonic valve leaflets with trace pulmonic regurgitation. A six-week course of intravenous vancomycin was completed and her convalescence was uneventful.

## 3. Discussion

Right sided endocarditis is an uncommon entity, accounting for only 5–10% of all infective endocarditis cases, but the majority involve the tricuspid valve [2, 3]. Isolated pulmonic valve endocarditis is rare with as few as 45 cases reported in patients with structurally normal hearts [4, 5].

The rarity of infection of the pulmonic valve compared to other cardiac valves has been attributed to the lower pressure within the right heart, lower incidence of congenital malformations or acquired valvular abnormalities, lower oxygen content of venous blood, and the differences in the endothelial lining and vascularization of the valve [6, 7]. Risk factors for pulmonic valve endocarditis include intravenous drug abuse, alcoholism, sepsis, central venous catheter infection, pacemaker implantation with lead infection, gonorrhea, dental extraction, bowel surgery, liver or renal transplantation, and colonic angiodysplasia [3, 6, 7].

Patients present primarily with typical features of endocarditis such as fever and lethargy; the pulmonic regurgitant murmur is often a late feature [5]. Patients with right sided endocarditis may also present with pulmonary symptoms such as cough, dyspnea, and pleuritic chest pain in the setting of septic emboli. Transthoracic echocardiogram can detect pulmonic valve vegetation, but transesophageal echocardiography has higher sensitivity and specificity [5]. The most common microorganisms reported are* Staphylococcus aureus*, coagulase negative staphylococci, and group B streptococci. In the 45 reviewed cases of pulmonic valve endocarditis in a normal heart* Staphylococcus aureus* is the most common causative organism [5].

The majority of patients with pulmonic or tricuspid valve endocarditis are managed conservatively. Right sided endocarditis has a better prognosis and is more likely to respond to medical therapy than infection of the mitral and aortic valves. Parenteral antibiotic therapy is generally administered for 4–6 weeks. Results of retrospective studies suggest that vegetation less than 1 to 2 cm long in right sided endocarditis usually responds to medical treatment [8, 9]. Surgery is indicated when there is persistent bacteremia despite appropriate antimicrobial therapy, progressive valve destruction and incompetence, locally invasive infection including abscess formation, or relapsing infection after completion of a full course of antibiotic therapy. It is recommended that infection with* Staphylococcus*, the presence of vegetation larger than 2 cm long, or cardiovascular instability should prompt consideration of early surgical intervention in patients with pulmonic valve endocarditis [10]. Surgical options include debridement of the infected area, vegetation excision with either valve preservation or valve repair or valve replacement. In cases where pulmonic valve replacement is unavoidable the use of a homograft or xenograft is recommended; however small studies suggest that mechanical valve prostheses in the pulmonic position may perform well [6, 11].

It is important to note that in our case the pulmonic vegetation was not reported on the preliminary echocardiogram performed at the outside hospital. The pulmonic valve is often not well visualized on TTE making the diagnosis of pulmonic endocarditis difficult. The pulmonic valve is best visualized in the RV outflow view. This view is obtained from the parasternal long-axis position by tilting the transducer superiorly and laterally (towards the left shoulder of the patient). Making an effort to visualize the pulmonic valve is crucial in patients in whom endocarditis is suspected.

Our patient's case is unique, in that no case has ever been reported in the literature of pulmonic valve endocarditis in a patient with sickle cell disease. Patients with sickle cell disease often require long term intravenous access which is a well-recognized risk factor for right sided endocarditis. Furthermore, the operative management of patients with sickle cell disease and endocarditis requires special consideration.

Surgical interventions, especially cardiac surgery, in sickle cell disease patients are associated with high morbidity and mortality [12–16]. Specific precautions are required during the perioperative period to minimize the surgical risks. Patients with sickle cell disease who require cardiac surgery are at risk of a fatal sickling crisis, induced by acidosis, hypothermia, hypoxia, or low flow states. Low oxygen tension and acidosis can induce sickling in patients on cardiopulmonary bypass and may trigger a crisis of profound magnitude [12–16]. While performing cardiac surgery, cardiopulmonary bypass, aortic cross clamping, low flow states, hypothermia, cold cardioplegia, and use of vasoconstrictive agents may predispose a crisis state [12]. To manage sickle cell patients, it is essential to maintain adequate blood flow and oxygen tension during cardiopulmonary bypass, avoid acidosis, control intra- and postoperative hemodynamic parameters, maintain hematocrit level of 20–30%, optimize electrolytes and blood gases, and minimize postoperative pain [12–16]. It is preferable to avoid hypothermia which can cause vasoconstriction and trigger a vasoocclusive crisis; however a number of studies have employed mild to moderate cooling without adverse effects [12–16]. Partial exchange transfusions during the pre- or intraoperative period are often recommended in cardiac patients to reduce the percentage of Hemoglobin S. In our case the patient did not require an exchange transfusion and normothermia was maintained to avoid vasoconstriction and sickling.

In general the implantation of prosthetic valves in patients with sickle cell disease is particularly problematic. Mechanical prostheses require systemic anticoagulation and may cause increased hemolysis and subsequent sickle cell crises. While bioprosthetic valves are the preferred type of prosthesis, the risk of infection of prosthetic material may expose these already vulnerable patients to recurrent endocarditis. Ideally these patients should undergo valve repair if surgically feasible. Furthermore it is recommended that any patient with infective endocarditis be evaluated and managed by a multispecialty heart valve team. In consultation with our heart valve team our patient underwent successful pulmonic valve repair and had an uncomplicated postoperative course. This unique case highlights the significance of performing a complete echocardiographic evaluation in any patient with suspected endocarditis and the importance of assembling a heart valve team to help guide treatment decisions in patients with infective endocarditis.

## References

[1] Cassling R. S., Rogler W. C., McManus B. M. (1985). Isolated pulmonic valve infective endocarditis: a diagnostically elusive entity. *The American Heart Journal*.

[2] Delahaye F., Goulet V., Lacassin F. (1995). Characteristics of infective endocarditis in France in 1991: a 1-year survey. *European Heart Journal*.

[3] van der Meer J. T. M., Thompson J., Valkenburg H. A., Michel M. F. (1992). Epidemiology of bacterial endocarditis in the Netherlands: I. Patient characteristics. *Archives of Internal Medicine*.

[4] Nishida K., Fukuyama O., Nakamura D. S. (2008). Pulmonary valve endocarditis caused by right ventricular outflow obstruction in association with sinus of valsalva aneurysm: a case report. *Journal of Cardiothoracic Surgery*.

[5] Schroeder R. A. (2005). Pulmonic valve endocarditis in a normal heart. *Journal of the American Society of Echocardiography*.

[6] Ranjith M. P., Rajesh K. F., Rajesh G. (2013). Isolated pulmonary valve endocarditis: a case report and review of literature. *Journal of Cardiology Cases*.

[7] Ramadan F. B., Beanlands D. S., Burwash I. G. (2000). Isolated pulmonic valve endocarditis in healthy hearts: a case report and review of the literature. *Canadian Journal of Cardiology*.

[8] Robbins M. J., Frater R. W. M., Soeiro R., Frishman W. H., Strom J. A. (1986). Influence of vegetation size on clinical outcome of right-sided infective endocarditis. *The American Journal of Medicine*.

[9] Hecht S. R., Berger M. (1992). Right-sided endocarditis in intravenous drug users: prognostic features in 102 episodes. *Annals of Internal Medicine*.

[10] Kang N., Smith W., Greaves S., Haydock D. (2007). Pulmonary-valve endocarditis. *The New England Journal of Medicine*.

[11] Waterbolk T. W., Hoendermis E. S., den Hamer I. J., Ebels T. (2006). Pulmonary valve replacement with a mechanical prosthesis. Promising results of 28 procedures in patients with congenital heart disease. *European Journal of Cardio-Thoracic Surgery*.

[12] Al-Ebrahim K. E. (2008). Cardiac surgery and sickle cell disease. *Asian Cardiovascular and Thoracic Annals*.

[13] Yousafzai S. M., Ugurlucan M., Al Radhwan O. A., Al Otaibi A. L., Canver C. C. (2010). Open heart surgery in patients with sickle cell hemoglobinopathy. *Circulation*.

[14] Métras D., Coulibaly A. O., Ouattara K., Longechaud A., Millet P., Chauvet J. (1982). Open-heart surgery in sickle-cell haemoglobinopathies: report of 15 cases. *Thorax*.

[15] Balasundaram S., Duran C. G., Al-Halees Z., Kassay M. (1991). Cardiopulmonary bypass in sickle cell anaemia. Report of five cases. *Journal of Cardiovascular Surgery*.

[16] Frimpong-Boateng K., Amoah A. G. B., Barwasser H.-M., Kallen C. (1998). Cardiopulmonary bypass in sickle cell anaemia without exchange transfusion. *European Journal of Cardio-thoracic Surgery*.

